# Impact of the COVID-19 Pandemic on Mental Health-Related Online Searches in Greece

**DOI:** 10.1192/j.eurpsy.2023.1698

**Published:** 2023-07-19

**Authors:** N. C. Xifaras, E. Roubou

**Affiliations:** National and Kapodistrian University of Athens, Athens, Greece

## Abstract

**Introduction:**

In recent years, there has been a well-documented increase in public perception of mental health (MH) matters, and in the related search for support by MH professionals. The emergence of the COVID-19 pandemic in 2020 has been a notable aggravating factor for MH around the globe, as well as in Greece.

**Objectives:**

Therefore, the goal of this study is to determine whether a significant change can be seen in the Google Search trends in Greece of specific terms related to MH after the start of the pandemic.

**Methods:**

Our data source was the Google Trends platform, which shows the relative volumes of Google Searches (relative search volumes, RSVs) happening in an area for the requested period of time, which in our case was January 2016-September 2022. Through a preliminary filtering of various search terms for data quality, we identified 6 for which the data were then statistically analysed as interrupted time series, to determine the significance of time and COVID-19 for the RSVs.

**Results:**

The terms analysed were “ψυχίατρος” (“psychiatrist”) (T1), “ψυχολόγος” (“psychologist”) (T2), “ψυχική υγεία” (“mental health”) (T3), “mental health” (T4), “κατάθλιψη” (“depression”) (T5) and “άγχος” (“anxiety”) (T6). Autoregressive integrated moving average (ARIMA) modelling and forecasting was used to account for the impact of previous months’ trends on each following month. The analysis showed a statistically significant relationship between the RSVs and time for all terms except T3 (p=.12). However, only T5 and T6 showed a significant change in the trend after March 2020 (p<.05); interestingly, they exhibited a downwards trend compared to their pre-COVID-19 volumes, after a peak in Spring 2020, which was not shared by the rest. T4, T5 and T6 RSVs were also correlated to the number of months since March 2020 (p<.05 for all).

**Conclusions:**

In conclusion, the impact of the pandemic on online search trends related to MH is limited for the majority of cases, and appears to have been time-bound to periods with intense extrinsic pressures (i.e. the emergence of an unknown disease and subsequent measures). More research is warranted to judge public sentiment towards and interest in the importance of MH and the true effects of COVID-19 on those; however, the constant rise of the search volumes is a positive sign for the recognition of the burden of MH issues.

**Disclosure of Interest:**

None Declared

